# Evaluation of engineered sorbents for the sorption of mercury from contaminated bank soils: a column study

**DOI:** 10.1007/s11356-020-12073-4

**Published:** 2021-01-09

**Authors:** Leroy Goñez-Rodríguez, Alexander Johs, Kenneth A. Lowe, Kimberly E. Carter, Frank E. Löffler, Melanie A. Mayes

**Affiliations:** 1 Environmental Sciences Division, Oak Ridge National Laboratory, 1 Bethel Valley Road, Oak Ridge, TN 37830 USA; 2 Department of Civil and Environmental Engineering, University of Tennessee, Knoxville, TN 37996 USA; 3 Department of Microbiology, University of Tennessee, Knoxville, TN 37996 USA; 4 Center for Environmental Biotechnology, University of Tennessee, Knoxville, TN 37996 USA; 5 Biosciences Division, Oak Ridge National Laboratory, Oak Ridge, TN 37830 USA

**Keywords:** Column study, Engineered sorbents, Mercury, Mercury immobilization, Sorption, Mercury source, Bank soil

## Abstract

As a global environmental pollutant, mercury (Hg) threatens our water resources and presents a substantial risk to human health. The rate and extent of immobilization of Hg^2+^ (hereafter, Hg) on engineered sorbents (Thiol-SAMMS®, pine biochar, SediMite™, Organoclay™ PM-199, and quartz sand as a control) was evaluated using flow-through column experiments. The effectiveness of the sorbents was based on (1) the percentage of Hg removed in relation to the total amount of Hg passing the sorbent column, and (2) the rate of Hg uptake compared to the nonreactive tracer bromide (Br^−^). All sorbents removed Hg to a certain extent, but none of the sorbents removed all the Hg introduced to the columns. Thiol-SAMMS showed the highest mean percentage of Hg removed (87% ± 2.9%), followed by Organoclay PM-199 (71% ± 0.4%), pine biochar (57% ± 22.3%), SediMite (61% ± 0.8%), and the control quartz sand (11% ± 5.6%). Thiol-SAMMS was the only sorbent to exhibit retardation of Hg in comparison to the conservative tracer Br^−^. For the remaining sorbents, Br^−^ along with low concentrations of Hg were eluted within the first 3 pore volumes, indicating limited retardation of Hg. Overall, removal of Hg by sorbents was substantial, suggesting that sorbents might be suitable for deployment in contaminated environments. High concentrations of DOM leaching from the soil columns likely influenced the speciation of Hg and inhibited sorption to the sorbents. Incomplete removal of Hg by any sorbent suggests that additional optimization is needed to increase efficiency.

## Introduction

Mercury (Hg) is a global pollutant that threatens our water resources and poses a significant risk to human health. Anthropogenic activities, including mining, industrial use, and fossil fuel combustion, have increased Hg flux into the atmosphere (Streets et al. 2011, 2017) and unintentionally increased Hg input to watersheds, lakes, and oceans. Mercury is converted in terrestrial and aquatic ecosystems between various forms, which include elemental Hg^0^, inorganic mercuric Hg^2+^ (dissolved and particulate), and organomercurials (e.g., methylmercury). Monomethylmercury (MeHg) is not typically released into the environment; it is formed through the conversion of inorganic Hg^2+^ to MeHg by anaerobic bacteria and archaea (Gilmour et al. 2013a; Parks et al. 2013; Podar et al. 2015). The conversion of Hg^0^ and Hg^2+^ to highly toxic MeHg in combination with bioaccumulation and biomagnification in aquatic environments leads to significant risks to environmental and human health (Clarkson 1998; Morel et al. 1998).

Engineered sorbents are used for the removal of heavy metals from industrial waste streams and in situ stabilization in contaminated subsurface environments. While remediation strategies using in situ sorbent amendments have been successfully demonstrated for many organic contaminants (Ghosh et al. 2011), large-scale applications of sorbents to remediate Hg remain limited. Studies have aimed to reduce the bioavailability of Hg for methylation and the bioaccumulation of MeHg (Gilmour et al. 2013b). Sorbents investigated include functionalized mesoporous silica (Fryxell et al. 1999), organocation-modified clay, brass (Wenke et al. 2016), and carbon-based materials such as activated carbon and biochar (Gilmour et al. 2013b; Gomez-Eyles et al. 2013; Liu et al. 2016, 2017; Paulson et al. 2018). Mineral or polymer scaffolds with high surface areas and functionalized with reactive ligands may effectively capture dissolved phase metals from solution (Chen et al. 1999; Crockett et al. 2016; Say et al. 2008).

Amending soils with activated carbon and biochar has been successful in field studies (Asasian et al. 2012; Beesley et al. 2010; Ghosh et al. 2011; Gilmour et al. 2013b, 2018; Gomez-Eyles et al. 2013). A year-long in situ amendment of SediMite in a salt marsh reduced MeHg concentrations, and to a lesser extent total Hg, in pore waters (Gilmour et al. 2018). Activated carbon and biochar materials produced from renewable, low-cost biomass feedstocks show low inherent toxicity (Janssen and Beckingham 2013; Jonkers et al. 2010) and effectively reduce the diffusive flux of contaminants into the water column, which also reduces bioavailability to organisms (Gilmour et al. 2013a). However, natural organic matter and sulfide species may compete with sorbents for the binding of Hg, including MeHg, which can reduce the effectiveness of a sorbent treatment. Natural organic matter is ubiquitous in aquatic environments and consists of a complex, heterogeneous continuum of particulates and high- to low-molecular weight species with different solubilities and variable reactivities towards Hg species (Aiken et al. 2011; Dong et al. 2011; Haitzer et al. 2003).

The impact of dissolved organic matter (DOM) on the effectiveness of sorbent materials considered for Hg remediation in soils and sediments is significant (Johs et al. 2019). Carbon-based materials (pine biochar, SediMite) and engineered materials such as functionalized clays (Organoclay PM-199 and Organoclay MRM) and mesoporous silica (Thiol-SAMMS) were evaluated using sorption isotherms, but the presence of DOM dramatically impeded Hg^2+^ sorption onto all sorbents (Johs et al. 2019). Differences in effectiveness between sorbents were also observed, e.g., the thiol-based Thiol-SAMMS under these conditions exhibited approximately twice the capacity of SediMite, which is an activated carbon-based sorbent. Organoclays and SediMite also released variable amounts of anions, specifically sulfate, which can enhance Hg^2+^ methylation by sulfate-reducing bacteria (Gilmour et al. 1992). Taking into consideration sorption efficiency, anion release, potential ecotoxicity, and cost considerations, carbon-based sorbents are promising candidates for the remediation of mercury-contaminated aquatic systems (Gilmour et al. 2013b; Johs et al. 2019).

Hardwood biochar was used to control the release of Hg^2+^ from sediments and floodplain soils in the South River watershed in Virginia (Paulson 2014). The sorption of Hg and MeHg was evaluated in laboratory experiments by connecting two columns in series (Paulson 2014). The “source column” contained Hg-bearing sediment and the second “treatment column” contained Cowboy Charcoal produced from pyrolysis. The treatment column was loaded with Hg, disconnected, and then subjected to clean influent solutions, and the extent of Hg release was measured. A decline in the dissolved Hg concentrations from the treatment column was observed over time, indicating strong efficacy of the sorbent for retaining Hg (Paulson 2014).

Engineered sorbents can reduce aqueous Hg concentrations and limit the formation of MeHg from aqueous systems. In this study, Hg-contaminated soil was leached to provide a source of aqueous labile Hg that retained properties of complexed Hg present in the environment. The leachate was then introduced into sorbent columns containing either Thiol-SAMMS, pine biochar, SediMite, Organoclay PM-199, or quartz sand as a control. The column method enables evaluation of both the rate and the extent of Hg sorption for each sorbent, and to our knowledge, no similar studies exist involving more than one sorbent. A nonreactive tracer was used to approximate the rate of Hg elution from the columns, and mass balance was used to calculate the extent of sorption. Mercury released in the soil column leachate was hypothesized to be captured effectively until the sorbent reached its maximum capacity.

## Materials and methods

### Mercury source

Mercury-contaminated soils (~ 22 kg) were collected from the creekbank of East Fork Poplar Creek (EFPC) in Oak Ridge, TN, USA, at a location 18.49 km (36° 00′ 24″ N, 84° 16′ 51″ W) upstream from the mouth of the creek (Brooks and Southworth 2011; Dickson et al. 2019; Southworth et al. 2013). The soil samples were mixed, homogenized, and stored at 4 °C until use. The total Hg concentration in the homogenized soil sample (determined by acid digestion as described below) was 1158 ± 534 mg Hg kg^−1^ dry weight (mean ± standard deviation, *n* = 2).

### Sorbents

Thiol-SAMMS consists of mesoporous silica support that is covalently modified with a self-assembled monolayer of thiol-functionalized organosilanes resulting in high surface area and high sorption capacity for rapid adsorption of Hg and other soft metal ions (Chen et al. 1999; Fryxell et al. 1999; Liu et al. 1998) (Table 1). Organoclay PM-199 (CETCO, Hoffman Estates, IL, USA) is a phyllosilicate clay modified with organocations and is commonly used for the remediation of hydrophobic organics. Biochar is produced by slow pyrolysis of Colorado pine softwood at 650 °C (BiocharNow, LLC, Loveland, CO, USA). SediMite (Sediment Solutions, LLC, Ellicott City, MD, USA) is a blend of 50% activated carbon along with bentonite clay, starch binders, and quartz sand pressed into pellets.

**Table 1:** Information about the evaluated sorbent materials

Class of materials	Sorbent	Type	Manufacturer
Silica-based	Thiol-SAMMS	Thiol-functionalized self-assembled monolayer on a mesoporous silica support	Steward Advanced Materials, LLC, Chattanooga, TN, USA
	IOTA	Quartz sand (control)	Unimin Corporation, New Canaan, CT, USA
Organo-clays	Organoclay PM-199	Functionalized bentonite-based clay	CETCO®, Hoffman Estates, IL, USA
Carbon-based	SediMite	Activated charcoal, bentonite, and sand as a weighting agent	Sediment Solutions, LLC, Ellicott City, MD, USA
	Biochar	Natural charcoal from Colorado pine converted by slow pyrolysis	Biochar Now, Loveland, CO, USA

### Column experiments

Laboratory-scale soil columns were used to mobilize Hg in leachate from the contaminated creekbank soils. The soils were packed into acrylic flow columns (Soil Measurement Systems, Tucson, AZ, USA) having an inner diameter of 7.5 cm and a length of 26.7 cm. A small amount of clean, acid-washed quartz sand (IOTA, Unimin Corp., New Canaan, CT, USA) was applied at the bottom (~ 45 g) to ensure even distribution of influent and the columns were packed with ~ 2-cm-thick layers of field-moist soil and gently compressed. Finally, a layer of quartz sand was added to the top (~ 45 g) of the column to prevent any soil from mobilizing with the column effluent.

After assembly, the soil columns were purged with CO_2_ from the bottom to displace the air inside the column and inhibit the formation of gas bubbles that can cause preferential flow (Xue et al. 1997). An artificial creek water (ACW) solution was designed to mimic the water chemistry of EFPC (Table 2). Soil column influent solutions were connected to a Deltec 3000 (Smiths Medical MD, Inc., St. Paul, MN, USA) modular infusion pump to introduce the ACW to the bottom of the column at a flow rate of 5 mL h^−1^ in order to saturate the column. After saturation, the flow rate was changed to 20 mL h^−1^ for the entire duration of the experiment.

**Table 2:** Chemical composition of artificial creek water (pH = 8)

Compound	mM
KNO_3_	0.048
NaNO_3_	0.068
Na_2_SO_4_	0.353
NaCl	0.418
Mg(NO_3_)_2_ · 6H_2_O	0.496
Ca(NO_3_)_2_ · 4H_2_O	1.036

Eight sorbents and two quartz sand (IOTA, Unimin Corp., New Canaan, CT, USA) columns were constructed with a similar design as the soil columns, only having an inner diameter of 2 cm and a length of 9 cm. The sorbent and sand columns were packed by slowly adding each sorbent or sand while tapping the columns to minimize the formation of void spaces. SediMite pellets are designed to disintegrate into fine powder over time when in contact with a liquid. Therefore, clogging was minimized by packing the SediMite columns with a 2-cm layer of quartz sand at the bottom, followed by a 4-cm layer of SediMite, mixed with sand to eliminate any void spaces, and then adding another 2-cm layer of sand. The top 1 cm of the column remained empty to allow the sorbent to expand. The sorbent and sand columns were saturated with ACW following the same procedure used for the soil columns.

**Fig. 1:**
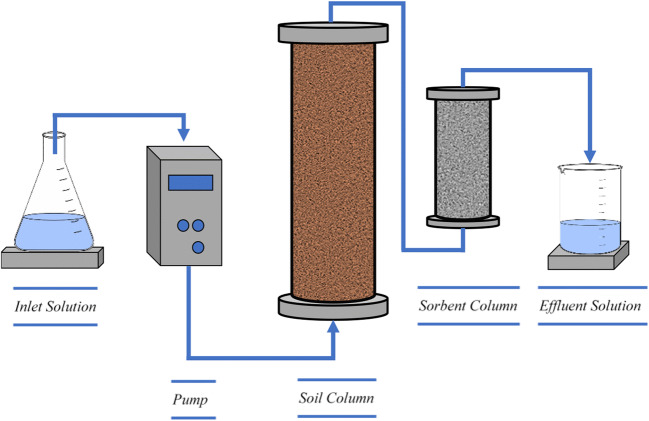
Experimental configuration, showing influent solution delivered via a pump into the soil column, and Hg-containing effluent exiting the soil column and entering the sorbent column, followed by effluent sample collection

After column assembly, packing with sorbents, saturation with ACW, and nonreactive tracer tests (see below), either sorbent or sand columns were attached to the effluent end of the soil columns, so that the Hg-contaminated effluent served as the source of Hg to the bottom of the sorbent or sand columns (Fig. 1). Flow direction was bottom to top in all columns in order to minimize entrapment of air and flow bypassing the media. After the experiments were completed, the columns were disassembled for the measurement of dry bulk density (*ρ*_*b*_) and porosity (ϕ). The soil, sand, and sorbents were dried in an oven at 70 °C for 2 days and the volume inside the column was measured to determine the *ρ*_*b*_ of each soil column. Porosity was calculated as follows:

1 $$ \phi =1-\frac{\rho_b}{\rho_P} $$

The density of the particles (*ρ*_*P*_) was assumed to be 2.65 g cm^−3^ for the soil columns. Since the *ρ*_*P*_ for the sorbent materials was not known, the following equation was used to determine porosity:

2 $$ \kern1.25em \phi =\frac{V_W}{V_T}\kern0.5em $$

The water volume (*V*_*W*_) was determined by weighing the column packed with the dry and saturated sorbent. The total volume of the column (*V*_*T*_) was calculated from the inner diameter and column height.

### Effluent sample collection

Three-way valves were placed at the effluent ends of both soil and the sorbent columns to facilitate sample collection. Samples were collected by diverting the flow into sample collection tubes of 7 or 50 mL, respectively. The 7-mL samples were collected daily for total Hg analysis. The 50-mL samples were collected approximately every 2 days for analyses of pH, dissolved organic carbon (DOC), specific ultraviolet absorbance at a wavelength of 254 nm (SUVA254), anions, and metals. The 50-mL samples were split into three aliquots: a 5-mL aliquot for pH and anions, a second 5-mL aliquot that was preserved with 0.2 mL of 12 M HCl for DOC and SUVA254 analyses, and a third 5-mL aliquot that was preserved with 0.015 mL of 16 M HNO_3_ for metals analyses. All samples were stored at 4 °C until analysis.

### Nonreactive tracer experiments

Nonreactive tracer experiments were conducted in the sorbent and sand columns using bromide (Br^−^) to quantify the basic hydraulic properties of the columns and to ensure that bypass flow along the walls of the columns was minimal (Mayes et al. 2003). Bromide was applied only to the sorbent columns and not to the soil columns because only the transport properties of the sorbent columns were needed (i.e., the sole purpose of the soil columns was to serve as a source of aqueous Hg). Bromide application was completed before the soil and sorbent columns were connected. The Br^−^ data are presented in relative pore volumes, which represents the volume of solution eluted compared to the total pore volume of the saturated pores in the columns; thus, one pore volume means that the amount of solution displaced is equivalent to the volume of water inside the sorbent column (Table 3). A three-way valve at the lower sorbent column boundary was used to make an instantaneous switch from the flowing ACW to a flowing solution of ACW with 10 mg L^−1^ Br^−^ added as KBr, using two different pumps each set to a flow of 20 mL h^−1^. Bromide was passed through each sand and sorbent column for 2 h, and effluent samples of 2 mL each were collected using a Spectra/Chrom CF-2 fraction collector (Spectrum Chromatography, Houston, TX, USA). After 2 h, the valve was switched back to the ACW to allow all of the applied Br^−^ to elute, while still collecting samples with the fraction collector. After the Br^−^ experiments were completed, the sorbent columns were attached to the outlet of the soil columns to allow Hg-containing soil column leachate to enter the sorbent columns. The initial breakthrough of both Hg and Br^−^ is presented as relative pore volumes so that their rates of elution can be directly compared to each other. The Br^−^ tracer is represented as relative concentration, i.e., the concentration of the tracer in the effluent over the initial concentration (C_Eff_/C_In_). We present the initial breakthrough of Hg and Br^−^ on the same figure to qualitatively compare the rates of Br^−^ and Hg breakthrough to each other.

**Table 3:** Convective-dispersive equation mobile-immobile (MIM) model parameters and outputs. Italicized font represents the inputs for the MIM transport model, and the regular font represents the outputs of the model

Material	Pore volume (PV) [cm^3^]	Porosity (θ)	Average pore velocity (ν) [cm h^−1^]	Pulse volume (T_0_)	Empirical first-rate coefficient (*α*) [h^−1^]	Dispersivity (*λ*) [cm]	Mobile fraction (*F*)	Retardation (*R*)
Sand	*14.3*	*0.51*	*12.59*	*2.96*	22.56	0.10 ± 0.03	0.94	1.55
ThiolSAMMS	*25.2*	*0.89*	*7.14*	*1.76*	0.19	0.30 ± 0.06	0.47	1.40
SediMite	*22.8*	*0.62*	*10.19*	*2.40*	0.74	0.54 ± 0.12	0.56	1.70
Organoclay	*21.4*	*0.67*	*9.45*	*–*	–	–	–	–
Pine biochar	*19.1*	*0.96*	*6.36*	*1.54*	43.07	0.34 ± 0.19	0.97	1.64

### Convective-dispersive transport equation

The convective-dispersive (CDE) equation (Parker and Van Genuchten 1984) was used to determine the hydraulic and geochemical transport parameters using the CXTFIT Excel code (Tang et al. 2010). The CDE transport model is defined (Parker and Van Genuchten 1984) as:

3 $$ R\frac{\partial C}{\partial T}=D\frac{\partial^2C}{\partial {X}^2}-v\frac{\partial C}{\partial X}-\mu C $$

where *C* refers to resident concentrations in the pore water [M L^−3^], *R* is the retardation factor [−], *D* is the dispersion coefficient [L^2 ^T^−1^], *v* is the average pore velocity [L T^−1^], *μ* is the first-order decay coefficient [T^−1^], *X* is the distance from the column inlet [L], and *T* is the time.

The transport equation can be reconfigured to represent non-equilibrium processes, such as diffusive exchange between mobile and immobile sites (MIM) or slow and fast reaction sites (2-site model). The MIM and the 2-site model are mathematically equivalent (Toride et al. 1999) in the formulation below:

4 $$ \beta R\frac{\partial {C}_m}{\partial T}=\frac{1}{P}\frac{\partial^2{C}_m}{\partial {X}^2}-\frac{\partial {C}_m}{\partial X}-\omega \left({C}_m-{C}_{im}\right) $$

5 $$ \left(1-\beta \right)R\frac{\partial {C}_{im}}{\partial T}=\omega \left({C}_m-{C}_{im}\right) $$

The aqueous concentrations in the mobile and immobile pores where the fluid is transported or retained (Geiser 2015) sites are *C*_*m*_ and *C*_*im*_, respectively. The mobile water fraction and the dimensionless mass transfer coefficient are represented by *β* and *ω* respectively, and *P* is the Peclet number [−] (Toride et al. 1999). The Peclet number (Eq. 6) is defined as:

6 $$ P=\frac{L\ v}{D} $$

where *v* [L T^-1^] is the mean pore water velocity and *L* is the column length [L]. The known parameters (inputs) for the CDE (Eqs. 7 and 8) are *v* and the pulse volume *T0* [−] of the Br^−^ influent (Tang et al. 2010) (Table 2). They are calculated from the effective porosity *θ* [−] (Eq. 2), Darcy velocity *q* [L T^-1^], pulse duration t_o_ [T], and *L*.

7 $$ v=\frac{q}{\theta}\kern0.5em $$

8 $$ {T}_o=\frac{q\ {t}_o}{\theta\ L}\kern0.5em $$

CXTFIT Excel estimates equilibrium and non-equilibrium parameters using a nonlinear least squares method (Tang et al. 2010). As a result of model fitting (Eqs. 9–12), we obtained dispersivity *λ* [L], *R*, *β*, and *ω*:

9 $$ D=\lambda\ v\kern0.5em $$

10 $$ {K}_d=\frac{\left(R-1\right)\ \theta }{\rho_b}\kern0.5em $$

11 $$ \alpha =\frac{\omega\ v}{\left(1-\beta \right)R\ L}\kern0.5em $$

12 $$ F=\frac{\beta \left(\theta +{\rho}_b\ {K}_d\right)}{\rho_b{K}_d} $$

Using the outputs of the MIM, we calculated the fraction of mobile water (*F*), the linear partition coefficient *K*_*d*_ [M T^−1^] which represents partitioning to the solid phase, and the mass transfer coefficient (*α*) [T^−1^] which represents the rate of exchange between the mobile and immobile fractions (Table 3).

### Solution analyses

The pH of the samples was measured using a benchtop pH meter Orion Dual Star (Thermo Scientific, Waltham, MA, USA). Dissolved organic carbon (DOC) was measured using a total organic carbon analyzer TOC-L CPH (Shimadzu Co., Kyoto, Japan). The baseline-corrected UV absorbance at a wavelength of 254 nm (A254) was measured using a Cary 60 UV-Vis spectrophotometer (Agilent Technologies, Santa Clara, CA, USA) and the specific UV absorbance (SUVA254) was calculated from the ratio of A254 and DOC concentration (Weishaar et al. 2003). Concentrations of anions were measured using a reagent-free ion chromatography system Dionex ICS-2100 (Thermo Scientific, Waltham, MA, USA). Dissolved metal concentrations (Al, Fe, Mn, and Si) were measured using an inductively coupled plasma mass spectrometer ELAN 6100 (PerkinElmer Sciex Instrument LLC, Norwalk, CT, USA).

Total mercury in samples was analyzed following EPA Method 1631 (USEPA 2002). Briefly, 200 μL of 2 M BrCl was added to 5 mL of sample and left to react overnight to quantitatively oxidize all mercury in the sample to the Hg(II) oxidation state. An aliquot was then added to 5 mL of 0.8% (w/v) stannous chloride in 0.5% HCl and purged with ultra-high purity N_2_. The emerging Hg^0^ was quantified by a cold vapor atomic absorption spectroscopy (CV-AAS) Zeeman effect Hg analyzer (RA-915+, Ohio Lumex Company, Inc., Twinsburg, OH, USA), which was calibrated with a set of Hg(NO_3_)_2_ standards (Brooks Rand Instruments, Seattle, WA, USA). The detection limit was 10 ng L^−1^. All samples were analyzed in duplicate.

Bromide concentrations were determined using a colorimetric assay adapted from Lepore and Barak (2009). Two stock solutions were prepared; 2.45 mM chloramine trihydrate and 1.63 mM phenol red (Sigma Aldrich, St. Louis, MO, USA). A buffer stock was prepared consisting of 0.5 M of sodium acetate, 0.5 M of glacial acetic acid, and 12.3 mM of ammonium acetate and adjusted to pH 4.6. The buffer stock and phenol red stock solutions were mixed immediately at a 1:1 (v/v) ratio before use. In a 1.5-mL microcentrifuge tube, 870 μL of the Br^−^ standard or sample was added to 65 μL of the phenol red-buffer mixture, followed by the addition of 65 μL of chloramine trihydrate. The mixture was allowed to react for 30 min. The solution was then transferred to a 1-mL cuvette and the absorbance at 590 nm was recorded using a UV-Vis spectrophotometer.

Statistical analyses were performed using the data analysis tools as implemented in Microsoft Excel. Two-sample *t* tests were conducted with a significance level of *p* = 0.05.

## Results

### Rate of Hg transport

From modeling the Br^−^ breakthrough curves with the MIM, all dispersivity (*λ*) values were < 1 cm, and all retardation factors (*R*) were < 2.0. Quartz sand and pine biochar had a mobile water fraction (*F*) of 0.94 and 0.97, respectively, and a relatively fast mass transfer coefficient *α* (> 20 h^−1^) (Table 3). ThiolSAMMS had an *F* of 0.47 and SediMite had an *F* of 0.56, and each had correspondingly low *α* (< 1 h^−1^).

**Fig. 2:**
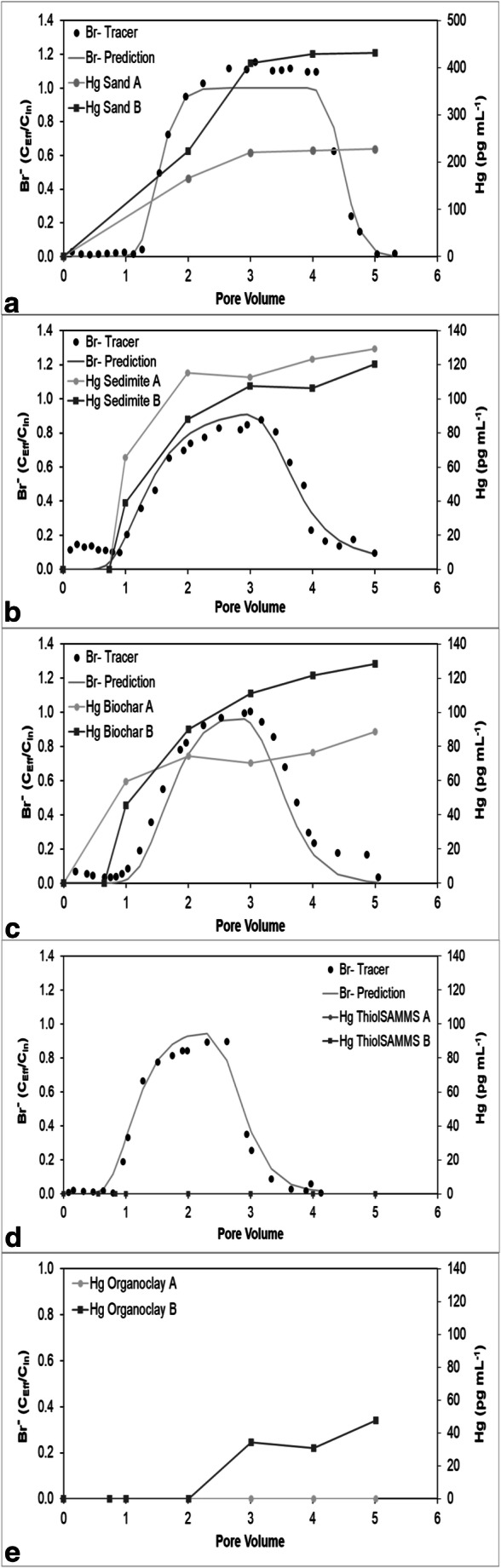
The mercury (Hg) and bromide (Br^−^) breakthrough curves for the following duplicate (A) and (B) columns (**a**) quartz sand, (**b**) SediMite, (**c**) pine biochar, (**d**) ThiolSAMMS, and (**e**) Organoclay PM-199. Data are plotted as relative pore volume (PV), which represents the volume eluted over the volume of wetted pores inside the column. C_Eff_ /C_In_ refers to the concentration of the Br^−^ in the effluent with respect to the concentration in the influent

In the quartz sand, SediMite, and pine biochar columns, Br^−^ and Hg breakthroughs are nearly simultaneous (Fig. 2a–c). Significant retardation of Hg was observed in the Thiol-SAMMS columns, with breakthrough occurring at 7 and 70 pore volumes in the two duplicate columns, neither of which are visible in Fig. 2d. Unexpectedly, Organoclay PM-199 was highly reactive to Br^−^ such that Br^−^ did not elute from the columns. The breakthrough of Hg occurred at 3 and 7 pore volumes in the Organoclay PM-199 sorbent (Fig. 2e).

### Extent of Hg removal

**Fig. 3:**
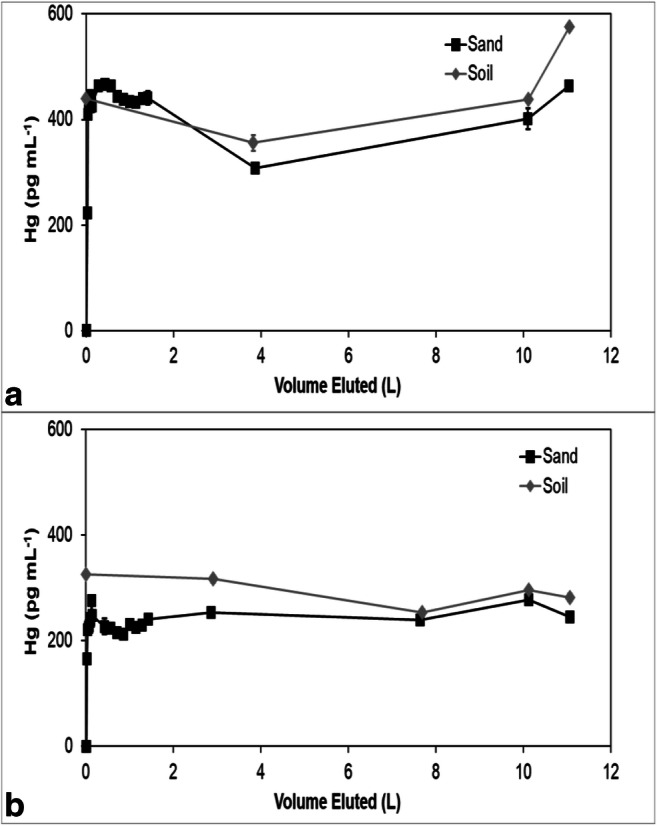
Total mercury (Hg) concentration in the effluents of duplicate (**a**) and (**b**) soil and sand columns

**Fig. 4:**
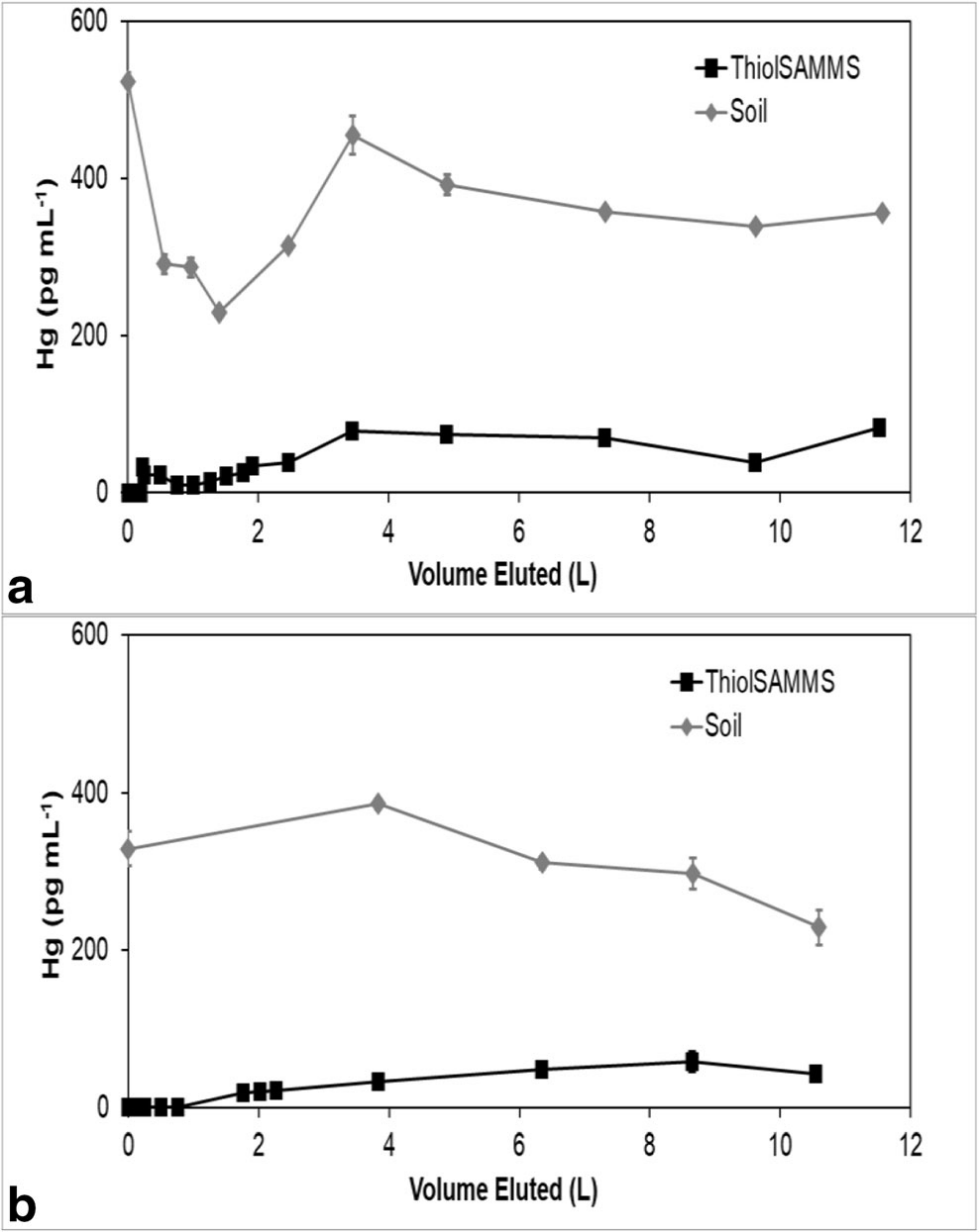
Total mercury (Hg) concentration in the effluents of duplicate (**a**) and (**b**) soil and ThiolSAMMS columns

**Fig. 5:**
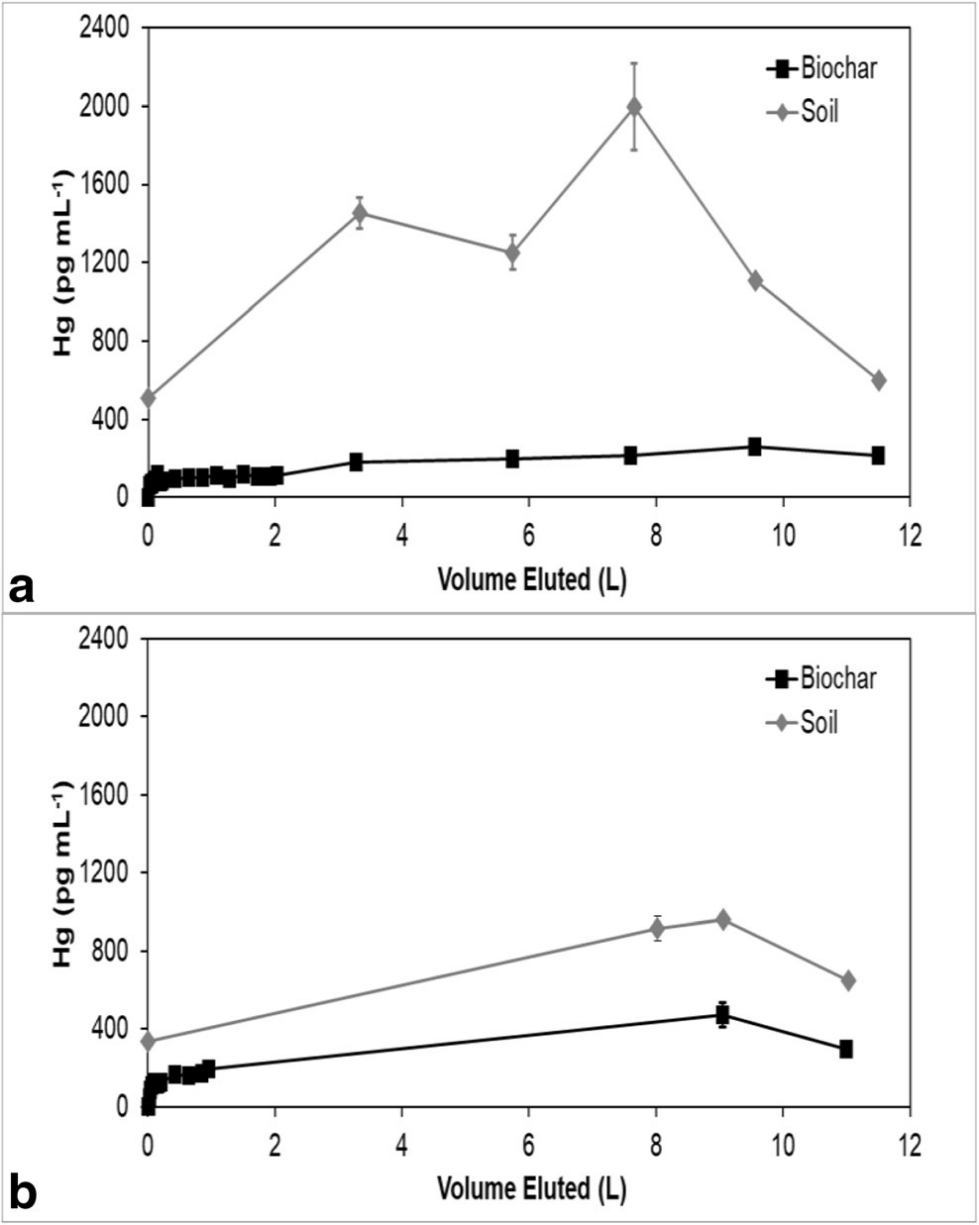
Total mercury (Hg) concentration in the effluents of duplicate (**a**) and (**b**) soil and pine biochar columns

**Fig. 6:**
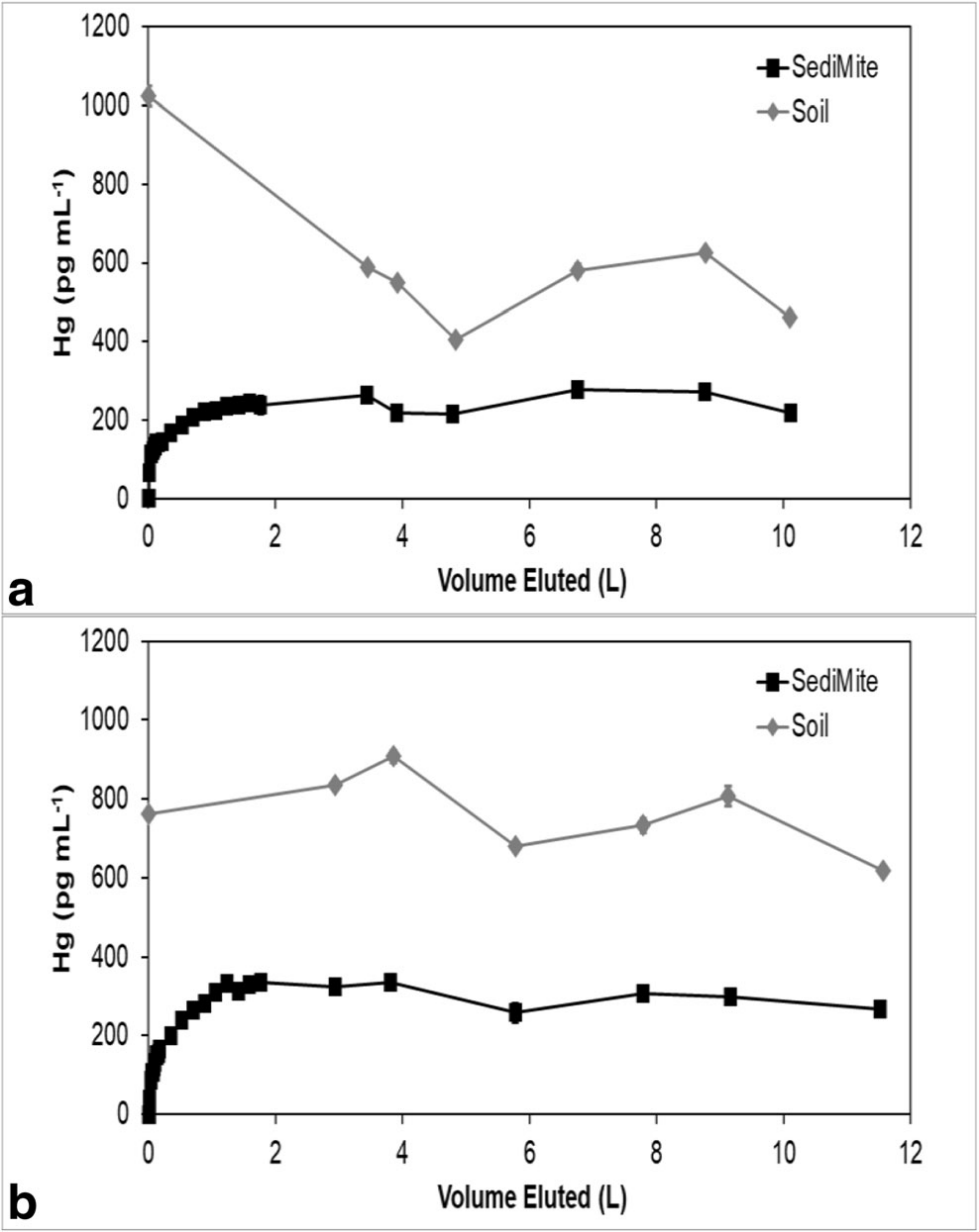
Total mercury (Hg) concentration in the effluents of duplicate (**a**) and (**b**) soil and SediMite columns

**Fig. 7:**
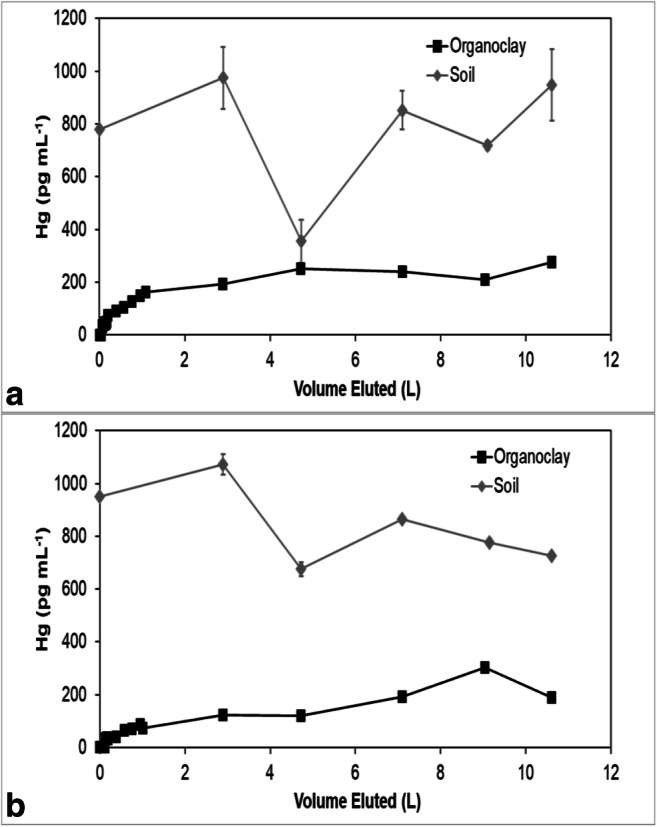
Total mercury (Hg) concentration in the effluents of duplicate (**a**) and (**b**) soil and Organoclay PM1-99 columns

Mercury concentrations in all the effluents eventually reached an approximate steady state, but the steady-state concentrations in the sorbent effluents were less than the effluent concentrations from the soils (Figs. 3, 4, 5, 6, and 7). The soil effluent curves represent the total Hg concentration that entered the sorbent columns over time. The sorbent (or sand) curves indicate the total Hg concentrations in the effluent of the sorbent (or sand) columns. The total mass of Hg sorbed by each sorbent (or sand) column is, therefore, the difference between the mass eluting from the soil column and the mass eluting from the sorbent (or sand) column (Table 4). For the sand columns, we observed only small differences between the Hg concentrations in the soil versus the sand column effluents (Fig. 3). The percentage of Hg sorbed to the two sand columns was generally low, only 15% and 7.5% respectively, which was in line with the expected low capacity of sand for retaining Hg (Table 4). This result is consistent with the similar timing of breakthrough of Hg and Br^−^ (Fig. 2a), whereas the breakthrough of Hg was rapid and occurred within the first two pore volumes for each of the sand columns.

**Table 4:** Mass balance of mercury (Hg) eluted from soil columns and sorbed by sorbent columns

Column	Material	Hg eluted from soil (μg)	Hg eluted from sorbent (μg)	Hg retained by sorbent (μg)	Hg retained by sorbent (μg Hg/g sorbent)	Hg retained (%)
A	Sand	3.23	2.74	0.50	0.02	15.4
B	Sand	4.49	4.15	0.34	0.01	7.50
A	ThiolSAMMS	8.65	2.16	6.49	0.93	84.7
B	ThiolSAMMS	7.64	1.17	6.47	0.96	88.8
A	SediMite	8.63	3.48	5.15	0.76	60.6
B	SediMite	9.86	3.77	6.09	0.84	61.7
A	Organoclay PM-199	17.4	7.37	10.0	0.57	70.7
B	Organoclay PM-199	19.9	5.71	14.2	0.60	71.3
A	Pine biochar	26.0	7.00	19.0	5.70	73.0
B	Pine biochar	15.7	9.21	6.51	1.86	41.4

Among all sorbent columns, Thiol-SAMMS (Fig. 4) removed the highest percentage of Hg, 85% and 89%, respectively (Table 4), which was consistent with the observed strong retardation of Hg (Fig. 2d). For the pine biochar columns, there was a difference in the effluent Hg concentrations between the two soil columns (Fig. 5). The average concentrations of Hg entering the pine biochar columns were 815 pg mL^−1^ and 467 pg mL^−1^, respectively, corresponding to a maximum concentration of 1996 pg mL^−1^ for column A and 961 pg mL^−1^ for column B (Fig. 5). The average percentage of Hg retained in the pine biochar columns was 73% and 41%, respectively (Table 4), where the column that removed 73% of the Hg was exposed to a higher Hg concentration. The percentage of Hg retained in the SediMite columns was 61% for both columns (Fig. 6) and 71% for Organoclay PM-199 (Fig. 7, Table 4).

### Chemistry of effluent solutions

**Fig. 8:**
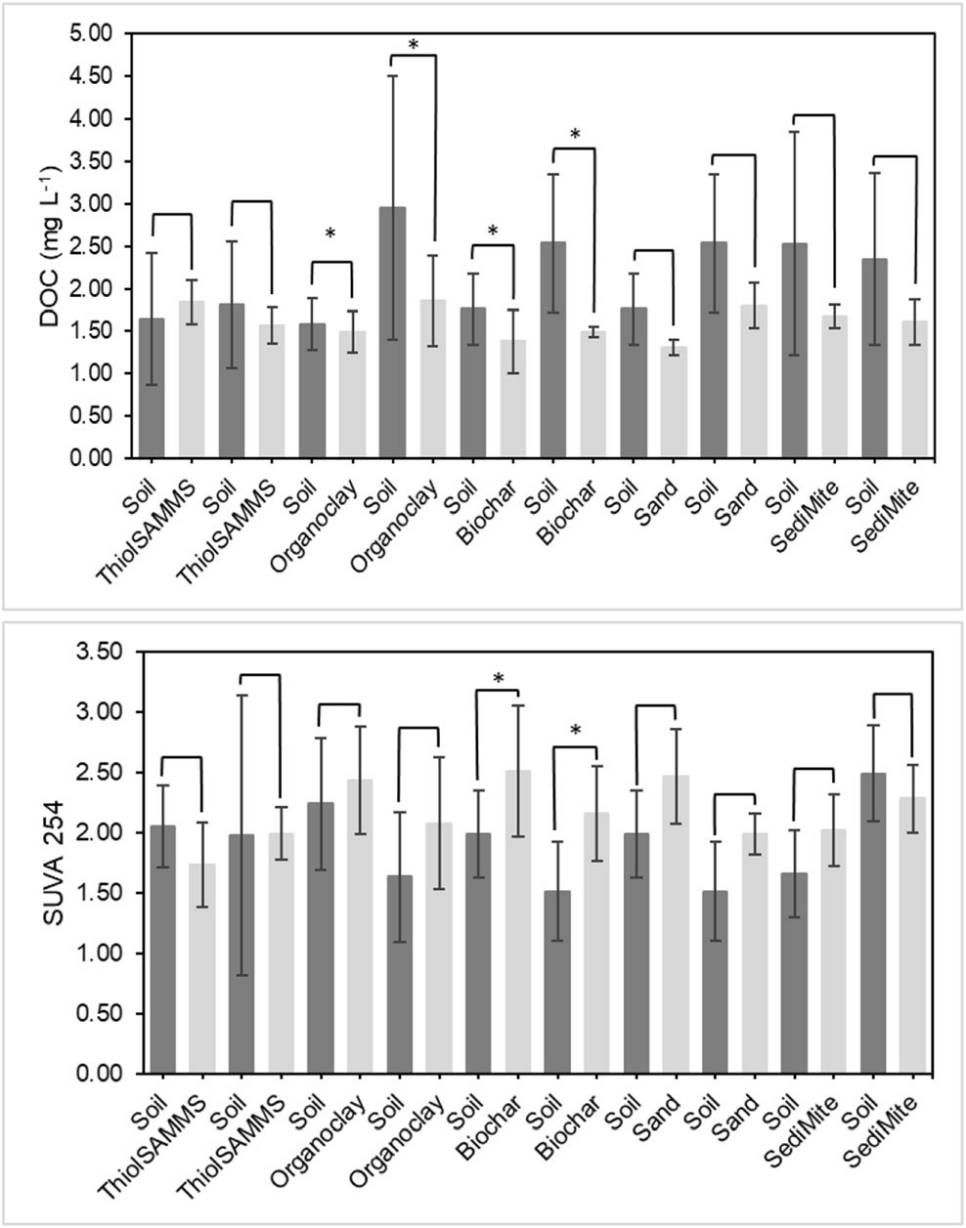
**a** Mean dissolved organic carbon (DOC) concentration in soil and sorbent effluents, with standard deviations, and **b** mean specific UV absorbance (SUVA) at 254 nm in soil and sorbent effluents, with standard deviations. Note that * indicates statistically significant differences, *p* < 0.05

The differences in DOC, pH, reactive metal, and anion concentrations between the soil and sorbent effluents were evaluated to gain insights into factors controlling Hg sorption in the sorbent columns. DOC concentrations were averaged over the entire experiment for each soil and sorbent column effluent pair (Fig. 8a). Small but statistically significant differences between the DOC concentrations in the effluents of the soil and sorbent columns were only observed for pine biochar and Organoclay PM199 columns (Table 5).

**Table 5:** *P* values obtained using *t* tests comparing dissolved organic carbon (DOC) concentrations and specific ultraviolet absorbance at 254 nm (SUVA254) in the effluents of soil and sorbent columns, where *p* < 0.05 represents statistical significance (*)

Column	Material	DOC	SUVA254
A	Sand (control)	0.426	0.131
B	Sand (control)	0.717	0.827
A	Thiol-SAMMS	0.102	0.242
B	Thiol-SAMMS	0.627	0.983
A	SediMite	0.513	0.256
B	SediMite	0.870	0.499
A	Organoclay PM-199	0.040*	0.059
B	Organoclay PM-199	0.422	0.343
A	Pine biochar	0.026*	0.026*
B	Pine biochar	0.003	0.000

The SUVA254 reflects the degree of aromaticity of DOC (Weishaar et al. 2003). Thus, it is an indicator of organic carbon quality and has been used to characterize Hg mobilization into surface waters from source areas (Burns et al. 2013). The mean concentration and standard deviation of SUVA254 in the soil and sorbent column effluents were measured and then averaged for each soil and sorbent column effluent pair (Fig. 8b). Pine biochar was the only sorbent that showed a small but statistically significant difference between soil and sorbent column effluents (Table 5). Overall, we observed a small increase in the SUVA254 values in the sorbent column effluents and a decrease in DOC concentrations over time, indicating increasing aromaticity with time. This increase suggests a small amount of preferential sorption of the non-aromatic DOM fraction in comparison to the aromatic fraction. The concentrations of other metals (Al, Fe, Mn, and Si), pH, and anions (Cl^−^, SO_4_^2−^, and NO_3_^−^) in the sorbent column effluents did not change in comparison to effluent from the soil columns (Goñez-Rodríguez 2018) (Table 6).

**Table 6:** Values obtained for metals (Al, Fe, Mn, and Si), pH, and anions (Cl^−^, SO_4_^2−^, and NO_3_^−^). Results are presented as a range, from the lowest to the highest value obtained for all the columns. BDL refers to below detection limit

			HPLC			ICP			
Column	Material	pH	Cl^−^ [mg/L]	NO_3_^−^ [mg/L]	SO_4_^2−^ [mg/L]	Al [μg/L]	Fe [μg/L]	Mn [μg/L]	Si [mg/L]
A	Sand	7.9–8.0	14.8–15.3	181–182	33.9–34.5	BDL	90.8	BDL	4.1
B	Sand	7.8–8.0	15.5	179	34.9	BDL	95.7	BDL	5.3
A	Thiol-SAMMS	–	15.3–15.5	177–191	34.2–34.7	BDL	BDL	BDL	5.5–6.2
B	Thiol-SAMMS	–	15.2–15.7	177–181	33.9–34.7	BDL	BDL	BDL	5.0–5.6
A	SediMite	7.9–8.0	14.9–16.4	181–195	33.6–36.7	BDL	89.8–103	BDL	4.7–5.1
B	SediMite	7.9–8.0	14.8–15.2	183–191	33.6–35.6	BDL	87.4–134	BDL	5.3–5.5
A	Organoclay	7.8–8.2	14.8–15.8	181–194	33.6–35.5	61.1–211	83.7–608	34.3–89.2	3.6–7.9
B	Organoclay	7.9–8.2	14.7–18.6	170–189	33.4–37.4	BDL	100–241	BDL	4.6–9.1
A	Pine biochar	7.9–8.1	14.8–15.8	181–193	33.5–35.2	BDL	94.2–285	BDL	3.9–6.0
B	Pine biochar	7.8–8.2	14.8–16.5	177–190	33.9–36.4	BDL	93.8–291	BDL	3.9–11

## Discussion

### Transport modeling and the rate of Hg transport

Bromide eluent curves were fit with the MIM to determine the hydraulic and geochemical parameters *λ, R, F*, and *α* (Levy and Chambers 1987). Dispersivity is a measurement of the rate of flow distribution through a system per unit length (Payne et al. 2008), and informs the extent of preferential flow, e.g., flow along the walls of the columns or bypassing of the sorbent media (Mayes et al. 2003). The values of *λ* were all < 1 cm (Table 3), which is substantially less than the column length of 9 cm, indicating minimal bypass flow. In soils, Br^−^ is usually a true conservative tracer, but engineered sorbents designed to retain solutes all exhibited some reactivity towards Br^−^. The retardation factor *R*, therefore, represented the reduction in the velocity of the mass of Br^−^ due to the reaction with the sorbent material. Thiol-SAMMS and SediMite showed the lowest and highest retardation factors, 1.40 and 1.70, respectively (Table 3). Thus, while Br^−^ was not entirely nonreactive (i.e., *R* = 1), its retardation with most sorbents was low. Only Organoclay PM-199 retained a substantial fraction of the added Br^−^, preventing determination of the hydraulic properties of the columns with this sorbent.

The fraction of mobile water *F* and the mass transfer coefficient *α* represent the proportion of “mobile” or “fast” reaction sites, and the exchange between these sites, respectively, in the MIM model (Tang et al. 2010). The model does not distinguish between the physical processes of mobile and immobile flow regions, and chemical processes of slow and fast reaction sites (Toride et al. 1999). Biochar and sand both had *F*~1 and a fast *α*, suggesting that the rate of transfer between the mobile and immobile fractions was very quick, indicating an approximate equilibrium (Tang et al. 2010). Thiol-SAMMS and SediMite both had *F* values near 0.50 and a slower *α* (Table 3), suggesting that there were two interacting regions having substantially different flow rates or reaction rates inside the columns and that the rate of exchange between the two regions was kinetically-limited (Mayes et al. 2003). For Thiol-SAMMS, this finding likely reflects the intraporosity of the functionalized monolayer emplaced on a mesoporous silica support bed, i.e., the design of the material involves both faster and slower reaction sites. SediMite was sandwiched in between two layers of quartz sand to minimize the clogging of the columns because the sorbent is designed to disarticulate in the presence of water. Therefore, finding relatively mobile and immobile regions in these two materials is consistent with the chemical and physical properties of the materials and the column design. In contrast, neither pine biochar nor sand has any properties that would be expected to result in multi-region flow or reactions, and no multi-region flow was observed. The results of the transport modeling suggest that the Br^−^ is a meaningful indicator of the sorption and transport characteristics of the sorbents, and therefore the Br^−^ breakthrough curves are a useful indicator of flow and transport in the columns for comparison to Hg.

In many cases, including pine biochar, SediMite, and sand, Hg breakthrough approximately coincided with Br^−^, indicating very limited retention of Hg. Unfortunately, much greater sample volumes were required for analysis of breakthrough curves for Hg compared to the Br^−^ tracer and the experiments did not result in a sufficient number of samples to use the MIM to calculate the retardation of Hg. Thus, our determination of the retardation of Hg is qualitative. However, for pine biochar and SediMite sorbents, Hg breakthrough occurred slightly before the Br^−^ breakthrough (Fig. 2b, c). The almost immediate elution of Hg from the sorbent columns illustrates that the sorbent could not retain all of the Hg to which it was exposed. Based on the modeled *λ* values, it is unlikely that the limited retardation of Hg is a result of bypass flow. Hg breakthrough for Thiol-SAMMS occurred after 7 and 70 pore volumes in the two duplicate columns (Fig. 4b), respectively. The results demonstrate that the sorbents tested in this study, with the notable exception of Thiol-SAMMS, did not effectively slow the rate of transport of Hg.

### Extent of Hg removal

The mass balance of eluted and sorbed Hg (Table 4) demonstrated substantial removal of Hg. None of the sorbents, however, were able to remove 100% of the applied Hg. Thiol-SAMMS showed the highest percentage removed, 85% and 89%. A higher variability between duplicates was observed for pine biochar, with 73% and 41% removed. This difference is likely due to heterogeneities in the soil columns which served as a source of Hg. The pine biochar column that removed a higher percentage of Hg was also exposed to a higher Hg concentration. Similar behavior was observed in another column study (Desrochers 2013), where the capacity of the sorbent for Hg uptake was also a function of the initial Hg concentration to which it was exposed. The columns containing quartz sand removed 15% and 7.5%, respectively, demonstrating that the sorbent columns were much more effective than the controls.

A column experiment using Hg-contaminated sediment from South River, Virginia, (USA) determined the potential of Cowboy Charcoal as a sorbent, and a much higher percentage of Hg was removed (> 98%) compared to the current study (Paulson 2014). The column size and geometry were similar to the present study, and the low *λ* values observed here do not suggest preferential transport. The higher flow rate in the present study could have imparted kinetic limitations on Hg reactions with the surface, but kinetic reaction rates on these sorbents are not yet available. The efficacy of sorbents is, however, influenced by particle size. In this case, the particle size of the pine biochar ranged from 0.65 to 3 mm, while the particle size of Cowboy charcoal was < 2 mm (Paulson 2014). The Cowboy charcoal most likely removed a higher percentage of Hg because the material had a smaller particle size representing a larger surface area for Hg retention, hence the difference in the effectiveness of the materials. In addition, differences in the Hg speciation between soil leachates from South River and EFPC bank soils could have influenced removal efficiencies, but the accurate determination of Hg speciation in soil leachates is very challenging and was not completed for either study.

### Solid-solution partitioning

The linear equilibrium partition coefficient (*K*_*d*_) is a commonly used parameter describing the effectiveness of sorbents and can be used to compare the amount of contaminant that can be removed under specific system conditions. While for many inorganic solutes partitioning reactions are controlled by changes in concentration and pH, the solid-liquid partitioning of trace level contaminants, such as Hg, is controlled by chemical speciation and the presence of competing ligands. The formation of strong complexes between Hg and DOM dominates Hg speciation in low sulfide environments and is a result of strong complexation of Hg with reduced sulfur (thiol) functional groups in DOM (Haitzer et al. 2002; Skyllberg 2008; Aiken et al. 2011; Dong et al. 2011). Thus, DOM can be considered a competing ligand, which influences Hg partitioning between solution and sorbents. High levels of DOM present in soil effluents may facilitate the mobilization of Hg and also limit the sorption of Hg species by sorbent materials. The role of mercury speciation and matrix effects on Hg removal efficiency was evaluated in a previous study (Johs et al. 2019). In a series of batch experiments, *K*_*d*_ values for the removal of Hg for the same sorbents used in the present study were determined by comparing the sorption of aqueous Hg(II) species to a well-defined Hg-DOM complex. This Hg-DOM complex was prepared by equilibrating Hg(II) with Suwannee River aquatic natural organic matter with known chemical composition and is representative of natural organic matter found across a wide range of environments (Green et al. 2015). This comparison of *K*_*d*_ values for Hg-DOM complexes with DOM-free aqueous Hg(II) species demonstrates that DOM limits the partitioning of Hg between solution and sorbents. Overall, *K*_*d*_ values for the Hg-DOM complex were up to two orders of magnitude lower compared to *K*_*d*_ values for aqueous Hg (II) species (Johs et al. 2019). The results from the column experiments in the present study are consistent with previous findings in batch experiments. For example, Thiol-SAMMS had the highest percentage of Hg removed from the sorbent columns (Table 4) and also exhibited the highest *K*_d_ in batch experiments (Johs et al. 2019). However, there are distinct differences between *K*_*d*_ and the total amount of Hg retained in column experiments. Based on the results from batch experiments with Hg-DOM, the *K*_*d*_ values for the sorbents can be ranked in the order of Thiol-SAMMS > SediMite > pine biochar > Organoclay PM-199 (Johs et al. 2019). In the present column study, the Hg retained by sorbents can be ranked in the order of Thiol-SAMMS > pine biochar ≈ Organoclay PM-199 > SediMite > > quartz sand. These results suggest that while the partitioning coefficient is an excellent indicator for the affinity of the sorbent for Hg, it is not a good indicator for the Hg sorption capacity of a sorbent.

### Mechanism of Hg removal

We evaluated differences in the chemical composition of soil and sorbent column effluents to examine potential mechanisms controlling Hg sorption. In comparing the soil and sorbent effluents, there were no significant changes in pH or the concentrations of major anions or cations, demonstrating that the sorbents did not alter the composition of the mobile phase by preferentially sorbing certain ions (Table 6). On average, most of the sorbent effluents showed a small decrease in the DOC concentration compared to the sorbent influents, but the difference was statistically significant only for pine biochar and Organoclay PM-199 (Table 5, Fig. 8a). The small decrease in DOC concentrations suggests very limited sorption of DOM. A general increase in the SUVA254 with a decrease in DOC in the sorbent column effluents of the pine biochar columns over time (Fig. 8b) suggests that pine biochar selectively removed some non-aromatic compounds from the DOM. The increasing SUVA254 values also correlate with increasing Hg concentrations in the effluent of the pine biochar columns, which might suggest that the sorption of Hg associated with the aromatic DOM fraction occurred to a lesser extent compared to the non-aromatic fraction. However, these differences were very small. We previously investigated the partitioning of DOM to the sorbents in batch experiments (Johs et al. 2019) and found that partition coefficients for DOC are generally one to two orders of magnitude lower compared to Hg partition coefficients. Therefore, Hg sorption in the soil effluents does not appear to be controlled by the direct sorption of DOM. The results for both the batch and column experiments consistently show that DOM limits Hg partitioning to the sorbents, but does not prevent it. Considering the large excess of DOM over Hg in solution and minimal observed changes in solution chemistry (anions, cations, DOC or SUVA254), we hypothesize two potential sorption mechanisms: (1) Selective sorption of Hg species, where the sorbent materials may sorb a subset of Hg species present in the soil effluents, or (2) a ligand exchange mechanism, where functional groups on the sorbent compete with existing Hg ligands in DOM for the binding of Hg. Future studies to identify Hg speciation and relevant functional groups on sorbent samples could help unravel Hg sorption mechanisms on a molecular level.

## Conclusions

The goal of this study was to evaluate the immobilization of Hg by engineered sorbents to reduce ambient concentrations in water leaching from contaminated soils. All the sorbents tested removed Hg to a certain extent (> 60%) from soil leachates, but none removed all of the Hg to which they were exposed. Few changes were observed in pH, DOC, SUVA254, or the concentrations of anions and cations in response to Hg sorption. While Thiol-SAMMS was the most effective sorbent of those tested, the present data suggest that a small fraction of the total Hg in the soil effluents remains in solution indicating that Hg speciation and DOM are important factors controlling removal efficiency. However, more research is needed to determine the molecular basis of Hg sorption mechanisms. Because of site-specific variabilities and our limited understanding of specific sorption mechanisms, additional optimization and in situ experiments should be performed to enable the use of sorbents to remove Hg from natural waters.

## Data Availability

Samples of the soils and the sorbents are available by request to the corresponding author. The data from this experiment will be made publicly available through a DOI at ORNL upon publication.
